# *Sargassum horneri* Extract Attenuates Depressive-like Behaviors in Mice Treated with Stress Hormone

**DOI:** 10.3390/antiox12101841

**Published:** 2023-10-10

**Authors:** Inhye Park, Jiwoo Kim, Minji Kim, Dong Wook Lim, Jonghoon Jung, Min Jung Kim, Junho Song, Suengmok Cho, Min Young Um

**Affiliations:** 1 Division of Functional Food Research, Korea Food Research Institute, Wanju 55365, Republic of Korea; p.inhye@kfri.re.kr (I.P.); k.jiwoo@kfri.re.kr (J.K.); 50036@kfri.re.kr (M.K.); dwlim@kfri.re.kr (D.W.L.); jonghoon@kfri.re.kr (J.J.); mjkim14@kfri.re.kr (M.J.K.); 2 Division of Food Biotechnology, University of Science & Technology, Daejeon 34113, Republic of Korea; 3 Department of Food Science and Technology, Institute of Food Science, Pukyong National University, Busan 48513, Republic of Korea; thdwnsgh1819@gmail.com (J.S.); scho@pknu.ac.kr (S.C.)

**Keywords:** depression, corticosterone, ERK-CREB-BDNF, HPA axis, neurotransmitter, *Sargassum horneri*

## Abstract

*Sargassum horneri*, a brown seaweed, is known for its various health benefits; however, there are no reports on its effects on depression. This study aimed to investigate the antidepressant effects of *S. horneri* ethanol extract (SHE) in mice injected with corticosterone (CORT) and to elucidate the underlying molecular mechanisms. Behavioral tests were conducted, and corticotropin-releasing hormone (CRH), adrenocorticotropic hormone (ACTH), and CORT levels were measured. A fluorometric monoamine oxidase (MAO) enzyme inhibition assay was performed. Neurotransmitters like serotonin, dopamine, and norepinephrine levels were determined. Moreover, the ERK-CREB-BDNF signaling pathway in the prefrontal cortex and hippocampus was evaluated. Behavioral tests revealed that SHE has antidepressant effects by reducing immobility time and increasing time spent in open arms. Serum CRH, ACTH, and CORT levels decreased in the mice treated with SHE, as did the glucocorticoid-receptor expression in their brain tissues. SHE inhibited MAO-A and MAO-B activities. In addition, SHE increased levels of neurotransmitters. Furthermore, SHE activated the ERK-CREB-BDNF pathway in the prefrontal cortex and hippocampus. These findings suggest that SHE has antidepressant effects in CORT-injected mice, via the regulation of the hypothalamic-pituitary-adrenal axis and monoaminergic pathway, and through activation of the ERK-CREB-BDNF signaling pathway. Thus, our study suggests that SHE may act as a natural antidepressant.

## 1. Introduction

Depression is a common mental disorder characterized by feelings of profound sadness, deprivation of interest, and decreased motivation [1,2]. Its occurrence is increasing worldwide [3] and has more than doubled in numerous countries since the onset of the coronavirus disease-19 pandemic. The current treatments for depression include monoamine oxidase (MAO), serotonin reuptake and noradrenaline reuptake inhibitors, as well as tricyclic antidepressants [4]. However, these drugs often lead to side effects such as insomnia, anxiety, xerostomia, severe weight loss [5], bleeding, and cardiovascular and gastrointestinal complications; these side effects often create obstacles to alleviating and preventing depression [4,6]. Furthermore, synthetic medications may sometimes lead to maladaptive responses, and individuals with treatment-resistant depression face a higher risk of relapse [7]. On the other hand, natural functional materials tend to be cost-effective, with fewer side effects compared to traditional antidepressants, and they are well-received by individuals struggling with depression due to their high adaptability [8]. Therefore, there is a need to develop functional ingredients that exhibit antidepressant effects as an alternative to drugs that have these side effects. 

Depression is often induced by chronic stress resulting from long-term exposure to stressful situations [9]. Under normal circumstances, the hypothalamic-pituitary-adrenal (HPA) axis regulates glucocorticoid levels such as corticosterone through a negative-feedback mechanism. However, in patients with depression, this regulatory process is disrupted [10]. Consequently, the corticotropin-releasing hormone (CRH), adrenocorticotropic hormone (ACTH), and corticosterone (CORT) levels are poorly regulated, leading to a continuous stress response that can trigger depressive symptoms and cognitive dysfunction [11,12]. In addition, MAO activity increases under sustained stress, accelerating the degradation of neurotransmitters, which are crucial for maintaining a normal mental state [13]. Moreover, the expression of brain-derived neurotrophic factor (BDNF) reduces during depression and stress, leading to decreased activation of the extracellular signal-regulated kinase (ERK)-cAMP response element-binding protein (CREB) signaling pathway, which reduces neurogenesis and neuronal function [14,15].

*Sargassum horneri*, a brown alga mainly found in the coastal waters of Korea, China, and Japan [16], is rich in phlorotannins, fucoxanthin, polysaccharides, and vitamins [17,18,19]. *S. horneri* has many biological properties, such as anti-inflammatory, antioxidant, and skin-protective effects [20,21]. Furthermore, Han et al. elucidated the neuroprotective effects of sargachromenol isolated from *S. horneri* [22]. Zhao et al. found that total sterols and β-sitosterol extracted from *S. horneri* exhibited antidepressant-like effects in mice, potentially mediated by increased brain neurotransmitters, norepinephrine and serotonin (5-HT) [23]. However, detailed studies addressing the antidepressant effects of *S. horneri* are yet to be reported.

In light of these findings, we hypothesized that *S. horneri* would exhibit antidepressant-like properties. To test this hypothesis, this study sought to explore the potential antidepressant effects of *S. horneri* ethanol extract (SHE) in a mouse model of CORT-induced depression. We further aimed to elucidate the underlying mechanisms, with a focus on the HPA axis, monoaminergic system, and ERK-CREB-BDNF signaling pathway.

## 2. Materials and Methods

### 2.1. Sample Preparation

*S. horneri* collected from Jeju Island (South Korea) in May 2022 were washed and lyophilized. Dried *S. horneri* were ground using a blender and extracted with 70% ethanol/water solution for 24 h at 25 °C. The extracts were subsequently filtered with filter paper (Whatman filter paper No. 2, Whatman, Maidstone, UK), concentrated by evaporation (Eyela, Tokyo, Japan), lyophilized, and stored at −70 °C until use. The yield of SHE was approximately 3.7 ± 0.1%.

### 2.2. HPLC Analysis

**Figure 1:**
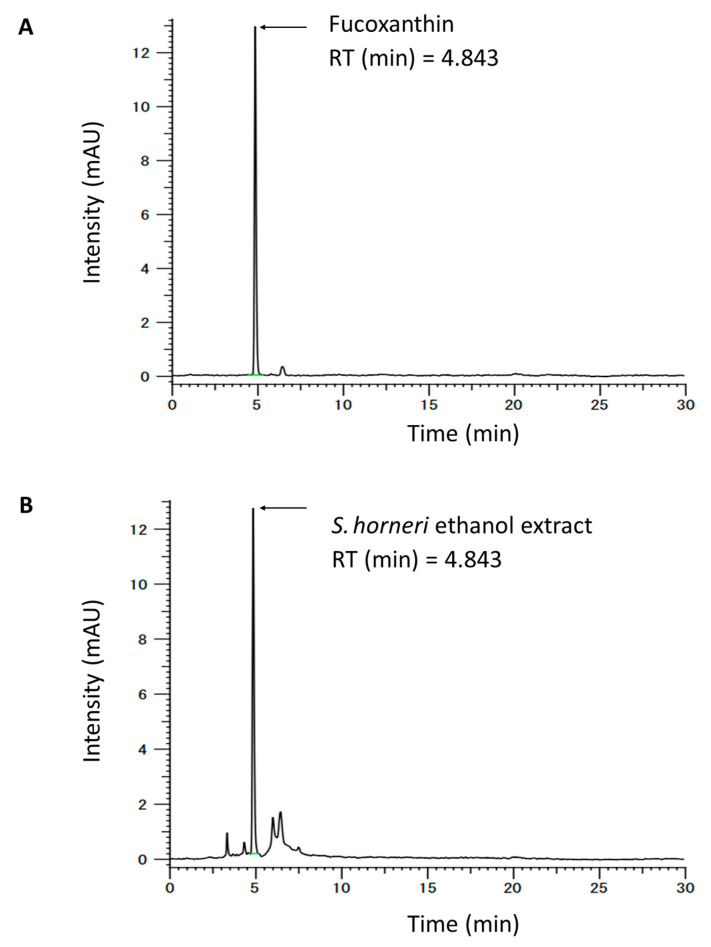
HPLC chromatogram of (**A**) fucoxanthin content in (**B**) *Sargassum horneri* ethanol extract (SHE).

The samples were analyzed using the HITACHI CM-5000 HPLC system (Hitachi Seisakusho Co., Ltd., Tokyo, Japan) and equipped with a CM-5110 pump and auto-sampler. For separation, a Supelco Discovery^®^ HS C18 RP column (5.0 μm, 4.6 × 250 mm) was used. The separation was processed with a methanol-acetonitrile solvent (7:3, *v*/*v*) with a 30 min running time and an injection volume of 5 μL. The flow rate was 0.7 mL/min, and each sample was detected at 450 nm. Briefly, SHE was dissolved in the mobile phase and filtered with a 0.22 μm membrane filter, and then the filtered sample was subjected to HPLC analysis. The regression equation and correlation coefficient (R^2^) of each standard curve were automatically determined using the HPLC system. The regression equation for fucoxanthin was y = 23927x + 6229.4 (R^2^, 0.9999). HPLC quantitative analysis was replicated four times. The concentration of fucoxanthin was 1.00 ± 0.02 (mean ± SD) mg/g extract, using the peak area in the standard chromatogram (Figure 1).

### 2.3. Animals and Treatments

Three-week-old male ICR mice (24–30 g) were purchased from KOATECH Animal, Inc. (Pyeongtaek, Republic of Korea). Mice were provided with food and water under controlled temperature (21 ± 2 °C), humidity (55 ± 5%), and a 12 h light–dark cycle. All animal experiments were approved by the Institutional Animal Care and Use Committee of the Korea Food Research Institute (IACUC number, KFRI-M-22005), and all experiments were performed in accordance with the Arrive guidelines 2.0.

**Figure 2:**
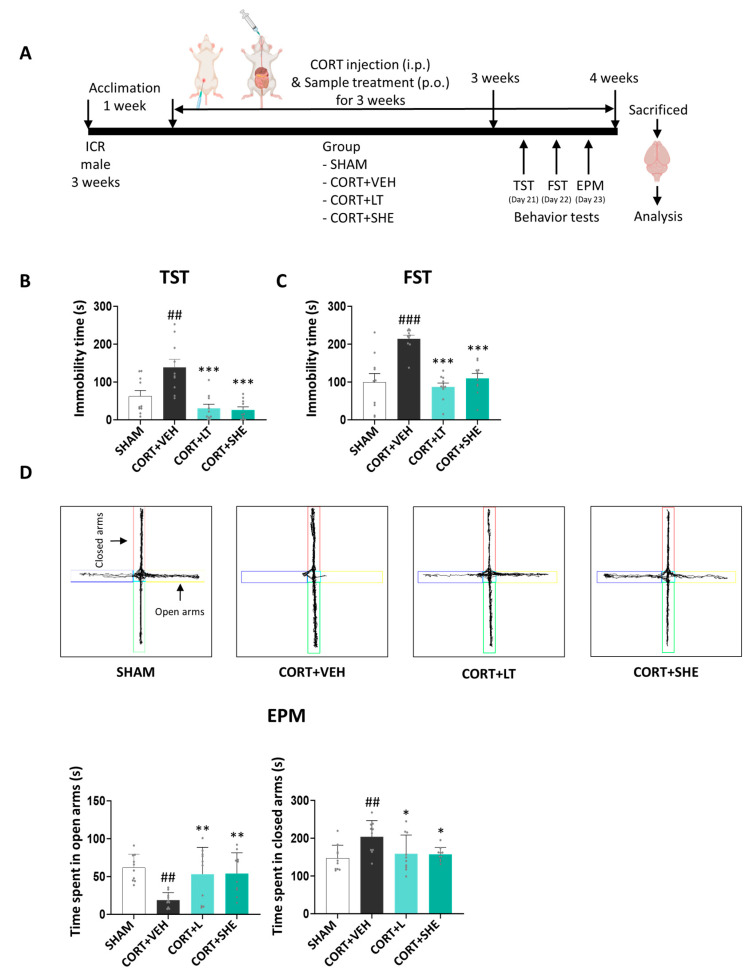
SHE mitigates depressive-like behaviors caused by corticosterone (CORT) injection. (**A**) Experimental procedure. (**B**,**C**) Immobility time in the tail-suspension test (TST) and forced-swimming test (FST). (**D**) The 5 min travel trajectory and the calculated time travelled in the elevated-plus-maze (EPM) test. ## *p* < 0.01, ### *p* < 0.001 vs. SHAM group: * *p* < 0.05, ** *p* < 0.05, *** *p* < 0.001 vs. CORT + VEH group. Closed arms: red and green colors; Open arms: blue and yellow colors.

After a 1-week acclimatization period, the mice were divided into four groups (n = 10 per group): (1) SHAM group (injected with vehicle (VEH) + VEH, per oral (p.o.) administration), (2) CORT + VEH group (injected with CORT + VEH (p.o.)), (3) CORT + LT group (injected with CORT + L-theanine (LT) for positive control group at 50 mg/kg BW (p.o.)), and (4) CORT + SHE group (injected with CORT + SHE 500 mg/kg BW (p.o.)). Mice were subjected to repeated intraperitoneal (i.p.) injections of CORT (40 mg/kg BW) for 3 weeks. The dosages were selected based on the relevant literature [24,25,26,27,28]. In this study, LT was used as the positive control. After 3 weeks, behavioral tests were performed, and the mice were sacrificed for analysis. Brain tissues were isolated and stored at −80 °C. The experimental scheme is shown in Figure 2A.

### 2.4. Behavioral Tests

#### 2.4.1. Tail-Suspension Test

The tail-suspension test (TST) was conducted following established protocols [29]. Animals were suspended 15 cm above the table using an adhesive tape that was placed approximately 1 cm from the tip of the tail. The immobility time was recorded for 6 min using the TST apparatus (BioSeb, Chaville, France).

#### 2.4.2. Forced-Swimming Test

The forced-swimming test (FST) was conducted following established protocols [30]. The mice were placed in the cylinders (height: 50 cm, diameter: 20 cm) and swam for 6 min. The immobility time was analyzed during the last 4 min of the test, excluding the initial 2 min. During 6 min, the immobility time was recorded and analyzed using SMART 3.0 software (Panlab SL, Barcelona, Spain).

#### 2.4.3. Elevated-Plus-Maze

The elevated-plus-maze (EPM) test was conducted in accordance with established procedures [31]. The EPM apparatus was elevated 60 cm from the floor, with two open arms (30 cm × 5 cm × 0.5 cm) and two closed arms (30 cm × 5 cm × 16 cm) connected by a central platform (5 cm × 5 cm). Each mouse was located on the center platform and allowed to acclimate for 1 min before commencing the test. Mice were positioned at the center of the maze and allowed to move freely for 5 min. Behaviors were recorded and analyzed using SMART 3.0 software (Panlab SL).

### 2.5. Measurement of Serum CRH, ACTH, and CORT Levels

Serum CRH, ACTH, and CORT levels were measured using commercial ELISA kits following the manufacturers’ instructions (CRH ELISA kit, MyBioSource, San Diego, CA, USA; ACTH and Corticosterone ELISA kits, Enzo Life Sciences, Farmingdale, NY, USA). Absorbance was measured at 450 nm using a microplate spectrophotometer (Jasco, Tokyo, Japan). All levels were calculated from a standard curve fitted to serial standards supplied by the manufacturers.

### 2.6. Measurement of MAO Activity

The fluorometric MAO enzyme inhibition assay was performed using a commercial kit (Thermo Fisher Scientific, Waltham, MA, USA). Briefly, human recombinant MAO-A or MAO-B enzymes (0.15 U/mL, Sigma-Aldrich, St. Louis, MO, USA), substrate (p-tyramine or benzylamine, 1 mM), horseradish peroxidase (1 U/mL), Amplex red reagent (200 μM) were mixed. SHE (1–200 μg/mL) was added to the mixture, and the mixture was incubated at 25 °C for 60 min while protected from light. Subsequently, fluorescence intensity was detected using a fluorescence microplate reader (Molecular Device, Sunnyvale, CA, USA) at excitation and emission wavelengths of 550 and 590 nm, respectively.

### 2.7. Measurement of Neurotransmitter Levels

Brain 5-HT, dopamine, and norepinephrine levels were determined following the manufacturers’ instructions (5-HT and dopamine ELISA kits, Abcam, Cambridge, MA, USA; norepinephrine ELISA kit, Feiyuebio, Wuhan, China). The sample absorbance at 450 nm was determined using a microplate spectrophotometer (Jasco).

### 2.8. Immunoblotting

The prefrontal cortex and hippocampal tissues were homogenized in a RIPA buffer supplemented with a protease-and-phosphatase inhibitor cocktail (Sigma-Aldrich). The brain homogenate was centrifuged (13,000 rpm, 10 min, 4 °C), and the supernatant was collected. The quantified proteins were separated using 10% sodium dodecyl sulfate–polyacrylamide gel electrophoresis and were transferred onto polyvinylidene fluoride membranes. After blocking with 5% skim milk (DB Bioscience, Detroit, MI, USA) for 1 h, membranes were incubated overnight at 4 °C with primary antibodies: p-ERK [4377S; Cell Signaling Technology (CST), Danvers, MA, USA], ERK (4695S; CST), p-CREB (9198S; CST), CREB (9104S; CST), BDNF (ab108319; Abcam), glucocorticoid receptor (GR; 12041S; CST), MAO-A (ab126751; Abcam), MAO-B (ab137778; Abcam), α-tubulin (3873S; CST), and β-actin (3700S; CST). All the primary antibodies were diluted at 1:1000. Following incubation with secondary antibodies, the membranes were visualized using an Enhanced Chemiluminescence reagent (Thermo Fisher Scientific). Relative densities were measured using ImageJ 8.0 software (National Institutes of Health, Bethesda, MD, USA).

### 2.9. Statistical Analysis

The data are expressed as the mean ± the standard error of the mean (SEM) and were analyzed using one-way analysis of variance (ANOVA), followed by Dunnett testing using Prism 9 (GraphPad Software, Inc., San Diego, CA, USA). Statistical significance was set at *p* < 0.05.

## 3. Results

### 3.1. SHE Improves Depressive-like Behaviors Caused by CORT Injection

Behavioral tests were conducted to analyze the antidepressant-like effects of SHE in mice induced by CORT injection (Figure 2B–D). In the TST and FST, the immobility time was significantly longer in the CORT + VEH group than in the SHAM group (TST: *p* < 0.05; FST: *p* < 0.001). However, these alterations were reversed by SHE administration to CORT-injected mice. In the EPM test, the time spent in open arms was reduced by approximately 3.3-fold in the CORT+VEH group, compared to that in the SHAM group (*p* < 0.01). However, SHE administration increased the duration of time spent in open arms (*p* < 0.01). Conversely, the time spent in closed arms was significantly increased by CORT injection in mice, but these effects were diminished by SHE administration, similar to that in the CORT + LT group. Our results indicated that SHE attenuated depressive-like behaviors in mice induced by CORT injection.

### 3.2. SHE Improves the Abnormal HPA Axis in Depressive Mice

**Figure 3:**
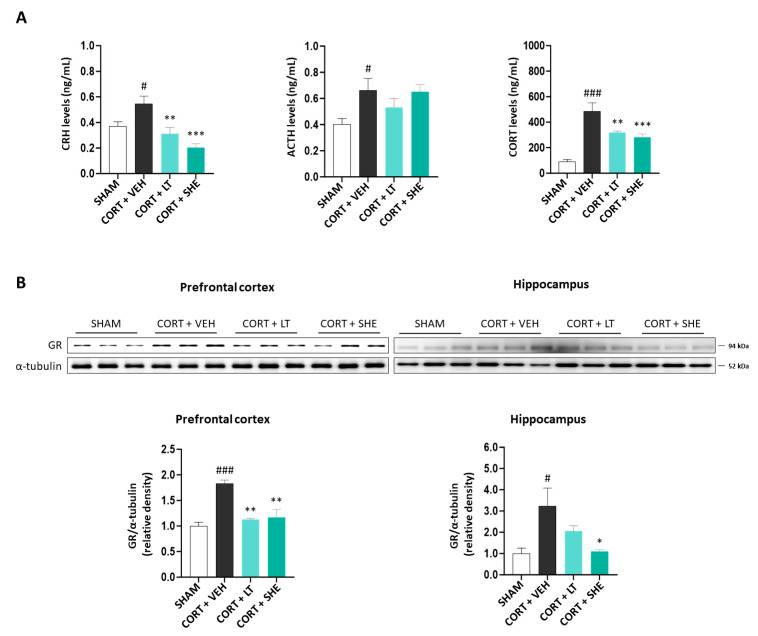
SHE improves the dysfunction of HPA axis in mice with CORT-injected depression. (**A**) Corticotropin-releasing hormone (CRH), adrenocorticotropic hormone (ACTH), and CORT levels in the serum of CORT-injected mice. (**B**) Glucocorticoid receptor (GR) protein expression in the prefrontal cortex and hippocampus of CORT-injected mice. # *p* < 0.05, ### *p* < 0.001 vs. SHAM group; * *p* < 0.05, ** *p* < 0.01, *** *p* < 0.001 vs. CORT + VEH group.

To investigate whether SHE modulates HPA axis dysfunction in mice induced by CORT injection, we measured serum CRH, ACTH, and CORT levels. CRH and CORT levels in the serum of CORT-injected mice were significantly higher than those in the SHAM group (*p* < 0.001); however, SHE administration decreased these hormone levels to levels similar to those in the CORT + LT group. Although the ACTH levels increased in the CORT + VEH group (*p* < 0.05), significant changes were not observed following SHE administration (Figure 3A).

GR regulates HPA axis activity during stress response [32]. Therefore, we measured GR protein expression in the prefrontal cortex and hippocampus. GR expression was higher in the CORT + VEH group than in the SHAM group (prefrontal cortex: 1.8-fold, *p* < 0.001; hippocampus: 3.2-fold, *p* < 0.05); however, this increase was mitigated by SHE administration (prefrontal cortex: 1.6-fold, *p* < 0.01; hippocampus: 2.9-fold, *p* < 0.05). Our findings suggest that SHE improves the HPA axis dysfunction in mice induced by CORT injection (Figure 3B).

### 3.3. SHE Prevents the Abnormal Monoaminergic System in Depressive Mice

**Figure 4:**
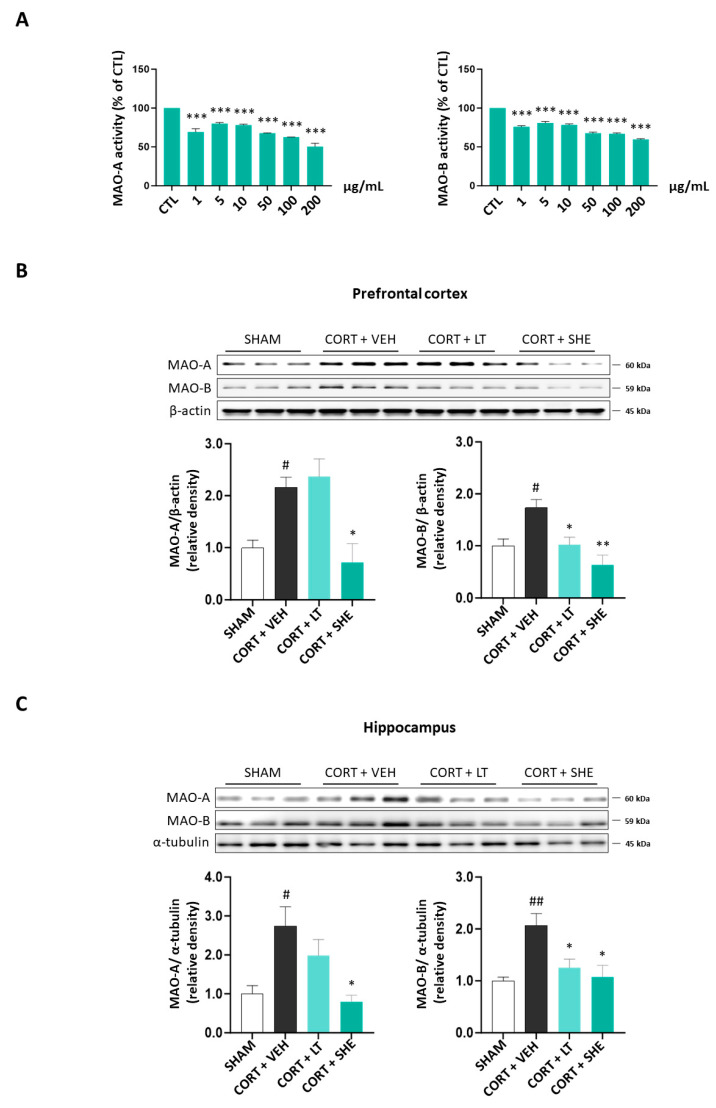
SHE improves the dysfunction of the monoaminergic system in CORT-injected depressive mice. (**A**) Monoamine oxidase (MAO)-A and MAO-B activities in in vitro assay. (**B**,**C**) MAO-A and MAO-B protein expressions in the prefrontal cortex and hippocampus of CORT-injected mice. # *p* < 0.05, ## *p* < 0.01 vs. SHAM group; * *p* < 0.05, ** *p* < 0.01, *** *p* < 0.001 vs. CORT + VEH group.

To assess whether SHE regulates the monoaminergic system in the CORT-injected depressive mice, we measured both MAO activity and its protein expression. First, we performed MAO activity assay in in vitro. The results showed that the MAO-A and MAO-B activities decreased by 1.7-fold and 2.0-fold at a concentration of 200 μg/mL SHE (MAO-A: 40.46% reduction; MAO-B: 49.58% reduction; Figure 4A).

Based on these results, we evaluated the levels of neurotransmitters, specifically 5-HT, dopamine, and norepinephrine, in the brains of CORT-injected mice. Although not significant statistically, a trend was identified whereby neurotransmitter levels decreased further in the CORT + VEH group than in the SHAM group. However, SHE administration led to increased levels of the neurotransmitters 5-HT, dopamine, and norepinephrine (Table 1).

Furthermore, in the prefrontal cortex and hippocampus tissues of CORT-injected mice, MAO-A and MAO-B protein expressions were significantly higher in the CORT + VEH group than in the SHAM group. However, SHE administration reduced the expression of MAO-A and MAO-B. These results suggest that SHE prevents a decrease in neurotransmitter levels in CORT-injected mice by modulating MAO expression (Figure 4B,C).

### 3.4. SHE Activates ERK-CREB-BDNF Signaling in the Prefrontal Cortex and Hippocampus of CORT-Injected Mice

**Figure 5:**
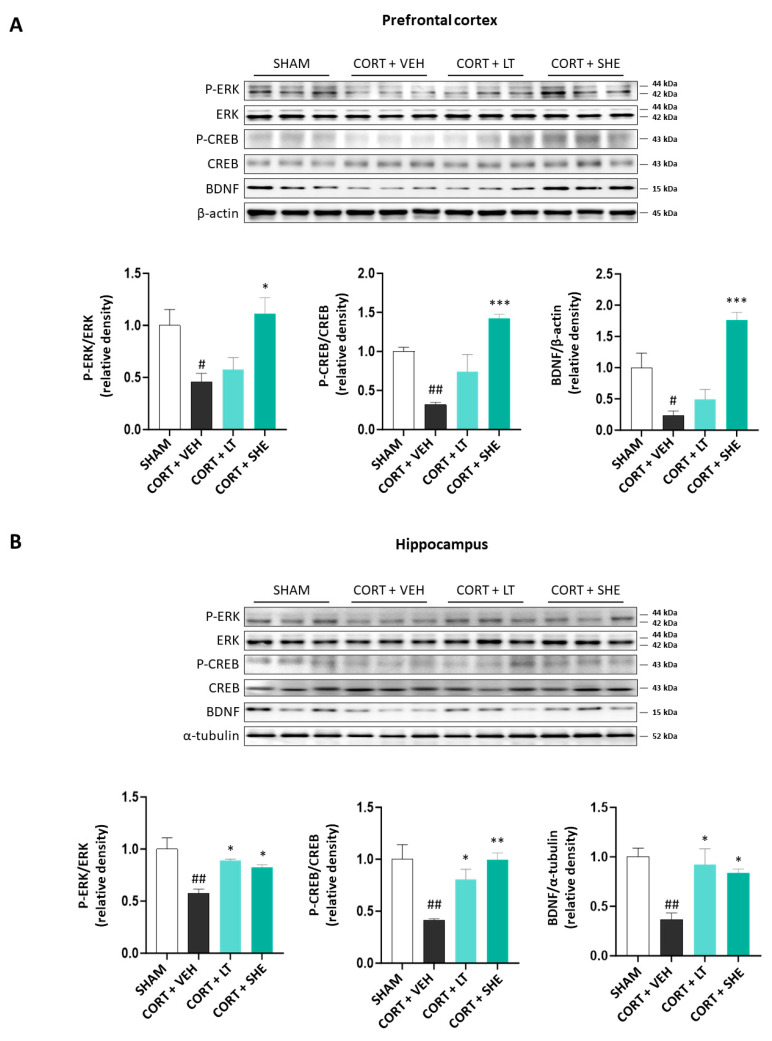
SHE upregulates extracellular-regulated kinase—cAMP response element binding—brain-derived neurotrophic factor signaling pathway in the prefrontal cortex (**A**) and hippocampus (**B**) of CORT-injected mice. # *p* < 0.05, ## *p* < 0.01 vs. SHAM group; * *p* < 0.05, ** *p* < 0.01, *** *p* < 0.001 vs. CORT + VEH group.

Recently, many studies have implicated the ERK-CREB-BDNF signaling pathway in depression pathology [14]. Hence, we evaluated the ERK-CREB-BDNF signaling pathway in the prefrontal cortex and hippocampus using immunoblotting. In the CORT + VEH group, the phosphorylation of ERK and CREB and the expression of BDNF were significantly lower than those in the SHAM group. However, SHE administration significantly increased the phosphorylation of ERK and CREB, and the expression of BDNF in CORT-injected mice. These results suggest that SHE activates the ERK-CREB-BDNF signaling pathway in the prefrontal cortex and hippocampus of CORT-injected mice (Figure 5).

## 4. Discussion

In the present study, we demonstrated the antidepressant effects of SHE on CORT-induced depressive mice. CORT, an adrenal hormone associated with stress, leads to sustained depressive behaviors such as low motivation or anhedonia [33]. The animal model of depression utilized in this study, induced by CORT injection, is widely used to verify the effectiveness of antidepressant drugs and phytochemicals [33,34]. The chronic CORT-treated rodent model is suitable for depression, as it is often used to assess mechanisms involved in depression. Consistent with previous research, our study revealed that mice injected with CORT exhibited depressive behavior distinguished as increased immobility time and anxiety. However, SHE administration significantly improved depressive behaviors following CORT injection. Yegdaneh et al. [35] reported that brown algae reduced immobility time in the FST and elevated appetite in a novelty-suppressed feeding test in a mouse model of depression induced by Bacillus Calmette-Guérin. Kim et al. [13] reported that an ethanolic extract of *Ishige foliacea* alleviated behavioral patterns in the FST and TST compared with a depression group. The hexane extract of *Sargassum plagyophylum* alleviates depressive-like behaviors by reducing immobility time in the FST [36]. Similar to previous research, SHE reduced the immobility time in the TST and FST and increased the time spent in the open arms in EPM. Our results showed that SHE mitigates depressive-like behavior in a CORT-induced depressed-mouse model.

Under stress, the HPA axis is activated, resulting in the release of CORT [37]. CORT binds to the GR and subsequently translocates to the nucleus, activating glucocorticoid-response elements to regulate the negative feedback process of the HPA axis [38,39]. However, normal regulation of the HPA axis is disrupted in depression, resulting in abnormal glucocorticoid overexpression, which further contributes to GR dysfunction [40]. Similarly, animal models of depression induced by CORT or restraint stress show high CRH and GR expression in chronically stressful situations [41,42]. Consistent with other studies, our results revealed that the serum’s CRH, ACTH, and CORT levels significantly increased in CORT-injected mice. In addition, the expression of GR in the brain tissues was noteworthy and increased in the CORT + VEH group. Drugs such as fluoxetine, ketamine, and xiaoyaosan reduce serum’s CORT concentrations and GR expression in animal models of chronic depression [43,44]. Similar to these antidepressants, SHE reduced GR expression and the concentrations of CORT and CRH, which were increased by CORT induction. Remarkably, unlike the concentrations of CRH and CORT, the ACTH concentration did not change after SHE administration. Previous studies measuring ACTH levels found no significant difference to the stress model at low concentrations of *Camellia euphlebia* and *Lilium davidii* extracts; however, a significant decrease was observed at high concentrations [45,46]. This suggests it is a dose-dependent response. We surmise that the singular SHE concentration in our experiments may not have reached the threshold to evoke discernible changes in ACTH levels. Given these findings, further experiments focusing on dose–response relationships are required. Nevertheless, because treatment with SHE reduced the levels of CRH and CORT, which were increased by CORT treatment, our results suggest that SHE may help alleviate HPA-axis dysfunction.

The absence of pleasant emotions and positive-affective states is a hallmark of depression, and these emotions are regulated by monoamine neurotransmitters [47]. Monoamine neurotransmitters, such as 5-HT, dopamine, and norepinephrine, are regulated by the MAO enzyme, which breaks them down. In mental disorders like depression and attention-deficit hyperactivity disorder, the monoaminergic system undergoes disruption through the activation of MAO, and subsequently resulting in accelerated degradation of neurotransmitters and impaired synaptic transmission [48,49,50]. Numerous antidepressants have been developed and used to inhibit MAO activity and prevent neurotransmitter degradation [51]. Poltyrev et al. [52] reported that antidepressants, such as ladostigil, inhibit MAO-A and MAO-B by more than 60% and significantly reduce stress-induced depressive-like behaviors. *Sargassum macrocarpum* ethanolic extract and sargachromanol, which is isolated from *Sargassum siliquastrum,* inhibit MAO-A and MAO-B activities [53,54]. Similar to other *Sargassum* species, SHE also inhibited MAO activity. MAO expression in the CORT-injected mouse brain tissues was significantly reduced by SHE. In a mouse model of depression, 5-HT concentrations reduced by CORT administration were increased by the antidepressant fluoxetine [55]. In our study, the administration of SHE led to a significant increase in neurotransmitter levels than in the CORT + VEH group. This effect was similar to that of antidepressants, where depressed mice showed a downward trend compared to the SHAM group. These results suggest that SHE may elicit the inhibitory effects of the MAO enzyme and prevent neurotransmitter degradation.

BDNF is a brain-neurotrophic factor that maintains neuronal function by promoting neuronal survival and differentiation [56]. BDNF binds to tropomyosin receptor kinase B and contributes to the activation of the ERK-CREB pathway through the phosphorylation of ERK and CREB. Upon activation, the ERK-CREB pathway is involved in signal transduction, cell growth, and cell death, contributing to the improvement of depressive symptoms [14]. Previous studies have shown reduced expression of p-ERK, p-CREB, and BDNF in the hippocampus of animal models of chronic unpredictable mild stress (CUMS) [57,58]. The antidepressant activity of fluoxetine was observed by stimulating the ERK-CREB-BDNF pathway in the CUMS mouse model [59]. Similar to antidepressants, SHE significantly increased p-ERK, p-CREB, and BDNF protein expression in the prefrontal cortex and hippocampus of the CORT-induced mouse model. As these results were similar to those observed in other brown algae [13], our results indicate that SHE plays a role in activating the ERK-CREB-BDNF pathway, thus contributing to the antidepressant effects in CORT-induced mice.

Fucoxanthin is a natural carotenoid found abundantly in edible brown seaweeds [60]. It is known to possess physiological and biological characteristics, such as antioxidative, anti-obesity, anticancer, antihypertensive, anti-inflammatory, antidiabetic, and neuroprotective properties [61,62]. A recent study by Pangestutie et al. [63], highlighted fucoxanthine’s potential to reverse oxidative stress and inflammation triggered by β-amyloid in BV2 microglia cells. This reversal was evidenced in the downregulated expressions of pro-inflammatory cytokines and a decrease in reactive-oxygen species formation. Furthermore, Jiang and colleagues [64] observed the efficacy of fucoxanthin in reducing the heightened immobility time of FST and TST after LPS treatment in mice. Specifically, a dosage of 200 mg/kg of fucoxanthin mitigated the overexpression of pro-inflammatory cytokines (IL-1β, IL-6, and TNF-α) and enzymes (iNOS and COX-2) in critical brain regions, implicating the modulation of the AMPK-NF-κB signaling pathway in its mechanism of action. In our studies, using HPLC analysis, we determined that the fucoxanthin content in SHE was 1.00 ± 0.02 mg/g extract. Given these findings, it is plausible to conclude that the fucoxanthin in the SHE likely contributed to its antidepressant effects.

However, this study had certain limitations. Firstly, because our experiments were conducted with a single concentration of SHE, it is necessary to verify the antidepressant effects of SHE at different concentrations in a CORT-injected mouse model. Secondly, although we observed the antidepressant efficacy of SHE in a CORT-induced depressive-mouse model, it is necessary to investigate other depressive models, such as social defeat stress- or chronic mild stress-induced depressive models. Thirdly, our study suggests that SHE has an antidepressant effect. Therefore, it is crucial to investigate and identify other active compounds in SHE that contribute to the antidepressant effect, even though fucoxanthin has already been identified. Finally, clinical trials are required to fully demonstrate the potential of SHE as a treatment for depression. Although the present research has focused on studying and analyzing the mechanisms involved in alleviating depressive-like behavior and the antidepressant effects of SHE in vitro and ex vivo, human clinical trials are required to determine the safety and effectiveness of the treatment.

## 5. Conclusions

In the present study, our findings demonstrate that SHE exhibits antidepressant-like effects in CORT-induced depressive mice by regulating CRH and CORT levels. The HPA axis normalized by SHE reduces GR overexpression. In addition, SHE restores the monoaminergic system by inhibiting MAO activity, which degrades neurotransmitters. Furthermore, the ERK-CREB-BDNF signaling pathways are phosphorylated and upregulated by SHE. Based on these results, this study has validated the antidepressant effects of SHE in a depressed-animal model, suggesting that it has potential as an antidepressant, which should be validated in future clinical trials.

## Figures and Tables

**Table 1:** Effects of *Sargassum horneri* ethanol extract (SHE) on the neurotransmitter levels in the brain.

pg/mg of Tissue	5-HT	Dopamine	Norepinephrine
SHAM	199.12 ± 22.54	34.32 ± 6.48	1138.31 ± 78.24
CORT + VEH	149.67 ± 13.56	20.23 ± 3.70	968.63 ± 74.15
CORT + LT	177.07 ± 31.42	39.34 ± 5.56	1023.68 ± 77.96
CORT + SHE	235.04 ± 20.05 *	48.96 ± 7.43 **	1253.98 ± 67.11 *

* *p* < 0.05, ** *p* < 0.01 vs. CORT + VEH group.

## Data Availability

The datasets are available from the corresponding authors on reasonable request.
